# Identification of risk factors for supra-therapeutic vancomycin trough levels in ventilator-assisted critical care patients based on integrated modeling and multi-criteria decision analysis

**DOI:** 10.1371/journal.pone.0324510

**Published:** 2025-05-23

**Authors:** Xi Cao, Bi-ting Zhu, Cai-peng Xie, Jing-yue Cai, Ding-guo Dong, Miao-ting Chen, Cheng-zhao Huang, Yong-chun Lin

**Affiliations:** 1 Department of Pharmacy, Central People’s Hospital of Zhanjiang, Zhanjiang, China; 2 Department of Pharmacy, Zhanjiang Potou District People‘s Hospital, Zhanjiang, China; 3 Comprehensive Second Ward of Critical Care Medicine, Central People’s Hospital of Zhanjiang, Zhanjiang, China; Children's National Hospital, George Washington University, UNITED STATES OF AMERICA

## Abstract

To explore the risk factors influencing vancomycin trough concentration ($C_{vg-min}$) overexposure in critically ill patients with mechanical ventilation and rank the factors, the medical records of 194 mechanically ventilated critically ill patients hospitalized from 12/10/2021–06/10/2024 were analyzed. Among 194 critically ill patients, 77.83% were male and 22.17% were female. Univariate analysis showed that oxygenation index (OI), activated partial thromboplastin time (APTT), urea nitrogen (UN), septic shock, heart disease, congestive heart failure (CHF), moderate/severe chronic kidney disease (CKD), etc. were statistically different (*P* < 0.05). And APTT, OI, CHF and moderate/severe CKD were statistically different in multivariate logistic regression (*P* < 0.05). The receiver operating characteristic (ROC) curve constructed for APTT and OI was 0.7779 (95% CI [0.708,0.848], *P* < 0.001), with a sensitivity and specificity were 72.99% and 71.93%, respectively. The consistency index (*CI*) and consistency ratio (*CR*) of analytic hierarchy process (AHP) was 0.0796 and 0.0885, respectively, which meets the consistency test standard. The contributions of APTT, OI, CHF and moderate to severe CKD to the overexposure of $C_{vg-min}$ were 0.0584, 0.1899, 0.1614 and 0.5902, respectively. The overexposure rates of $C_{vg-min}$ in patients with moderate/severe CKD and CHF were 95.12% and 95.23%, respectively. With regard to OI, when the cutoff value of OI was less than 245, the $C_{vg-min}\;$overexposure rate was 83%, otherwise, the overexposure rate was 60.97%. The risk factors for excessive exposure of $C_{vg-min}$ in critically ill patients with mechanical ventilation were ranked as follows: moderate/severe CKD > OI > CHF > APTT.

## Introduction

Vancomycin is a glycopeptide antibiotic widely used for treating multi-resistant gram-positive bacterial infections, especially those involving methicillin resistant *Staphylococcus aureus* (MRSA) [1–6]. However, it is reported that patients with MRSA infection treated with vancomycin suffered from acute kidney injury (AKI) due to vancomycin overexposure [7–9]. Therefore, the therapeutic drug monitoring (TDM) implementation of vancomycin is necessary, so as to ensure the safe and effective use of the drug, and avoid excessive or low concentrations leading to sub treatment levels, treatment failure, and toxicity [4].To promote the TDM implementation of vancomycin, the Division of Therapeutic Drug Monitoring of the Chinese Pharmacological Society published an initial clinical practice guideline in 2015 [10]. With concerns about the enhanced resistance of bacteria to vancomycin, clinical practice guidelines have recommended the higher therapeutic levels of vancomycin [11]. Specifically, the area under the curve/minimal inhibitory concentration (AUC/MIC) of vancomycin is regarded as the most accurate reflection of bacterial eradication for patients infected with *Staphylococcus aureus* [12,13]. Unfortunately, the MIC is not generally available for microbial isolates, or for presumed pathogens in the clinical setting. Since AUC is often unavailable, trough levels of antibiotics are commonly used as proxies, though this may be inaccurate [14]. Optimizing vancomycin therapy in the intensive care settings has been the focus of numerous studies in the past [15–22]. To reduce the occurrence of therapeutic failure and the development of resistant pathogens, a series of guidelines have been developed [16]. Notably, the guideline indicates the particular vulnerability of critically ill patients, where unpredictable creatinine clearance fluctuations significantly complicate the predictions of $C_{vg-min}$. This pharmacokinetic variability, combined with the altered drug metabolism in critical illness, increases the risk of clinically significant nephrotoxicity, presenting dual challenges in balancing therapeutic efficacy and patient safety [10]. However, clinical observations reveal a significant prevalence of $C_{vg-min}$ overexposure among critically ill patients undergoing mechanical ventilation.

This study aimed to investigate the risk factors contributing to this pharmacological phenomenon and systematically examine its clinical implications through retrospective comprehensive analysis of clinical medical records by univariate screening, multivariate logistic regression modeling and AHP among critically ill patients undergoing mechanical ventilation.

## Materials and methods

### Patients and data collection

Clinical observations indicate that numerous mechanically ventilated critically ill patients experience excessive $C_{vg-min}$ exposure. To find out the cause of this phenomenon, we began to apply for relevant research and retrospectively collected data of these patients from October to December 2024 (their hospital stay was from 12/10/2021–06/10/2024) for analysis. Therefore, 194 critically ill patients who were admitted from 12/10/2021–06/10/2024 in the Central People’s Hospital of Zhanjiang were enrolled in this study. This study was approved by the Ethical Committee for Research (approval number: KYYS-2023–87) and carried out in complete agreement with the pertinent version of the Declaration of Helsinki and all the other relevant regulations.

All volunteers provided written informed consent before participation in the study. The inclusion criteria were as follows: (1) those aged ≥18 years; (2) requiring vasoactive drugs to maintain blood pressure; (3) vancomycin was used and the time of administration and sampling were recorded accurately; and (4) undergoing mechanical ventilation. The exclusion criteria were shown below: (1) those having serious adverse drug reactions; and (2) un-standard samples. Vancomycin was uniformly infused for 1 ~ 3 h, and blood samples were collected 0.5 h before (trough concentration) and 1 h after the end (peak concentration) of administration. Other data such as creatinine clearance were recorded based on the actual test date. The clinical data of 194 critically ill patients were collected and sorted out by using Microsoft Office Excel, and later analyzed by single factor analysis, regression analysis and AHP with SPSS (IBM SPSS Statistics 21), MATLAB (Matlab2012a) and other software.

AHP is a decision analysis method to solve multi-objective complex problems. This method combines quantitative analysis with qualitative analysis, uses the experience of decision makers to judge the relative importance between the criteria for measuring whether the goal can be achieved, reasonably gives the weight of each criterion for each decision-making scheme, and utilizes the weight to find the order of the advantages and disadvantages of each scheme. AHP is more effectively applied to topics that are difficult to be solved by quantitative methods. The method includes constructing judgment matrix, hierarchical single ranking and consistency test, hierarchical total ranking and one-time test. Consistency check steps of judgment matrix:

(I) Calculating consistency indicators (*CI*)


$$\begin{array}{c} CI=\frac{\lambda_{\text{max}}^{\text{'}}\text{ - n }}{\text{n - 1}}\; \end{array}$$ (1)

(II) Finding the corresponding average random consistency index (*RI*)

The *RI* value is to construct 500 sample matrices by the random method: numbers from 1 ~ 9 and their reciprocals are randomly extracted to construct a positive reciprocal matrix, then the average value of the maximum eigenvalue ${\text{λ}}_{\text{max}}^{\text{'}}$ is obtained and defined as:


$$\begin{array}{c} RI=\frac{\lambda_{\text{m}ax}^{\prime }\;-\text{n}}{\text{n}-1} \end{array}$$ (2)

This is the Saatty rule.

(III) Calculating the consistency ratio (*CR*)


$$\begin{array}{c} CR=\frac{CI}{RI} \end{array}$$ (3)

When *CR* < 0.10, it is considered that the consistency of the judgment matrix is acceptable, otherwise the judgment matrix should be properly corrected.

### Statistical analysis

Statistical analyses included Student’s t-tests for mean comparisons and logistic regression for multivariate analysis, with a significance threshold of *P* < 0.05.

## Results

### General clinical data of patients

A total of 194 critically ill patients undergoing mechanical ventilation were included, including 151 males (77.83%) and 43 females (22.17%). The age, body weight and APAPHE II score of the patients were 63.86 ± 14.39, 64.22 ± 11.80 and 20.80 ± 6.64, respectively. The proportion of critically ill patients requiring vasoactive agents was 59.79%; to be specific, the proportions were 64.23% in the vancomycin overexposure group and 49.12% in the compliant group, and there was no statistical difference between the two groups (*P *> 0.05). The baseline data of the enrolled patients are shown in Table 1.

**Table 1:** Demographic, Laboratory Variables and clinical data.

Characteristic		Total (194)	Compliant group (57)	Overexposure group (137)	*P*-value
Age, years		63.86 ± 14.39	59.79 ± 13.54	65.55 ± 14.45	0.791
Male, n (%)		151(77.83%)	50(87.72%)	101(73.72%)	0.051
Weight, kg		64.22 ± 11.80	67.33 ± 12.08	62.93 ± 11.48	0.931
Albumin(g/L)		31.59 ± 6.09	32.34 ± 6.02	31.27 ± 6.12	0.996
APAPHE II score		20.80 ± 6.64	19.42 ± 6.81	21.38 ± 6.51	0.539
WBC (x10^9^/L)		13.70[9.42, 18.64]	14.30[10.75, 19.50]	13.10[8.68, 18.61]	0.242
PLT (x10^9^/L)		176.00[82.50, 281.00]	185.00[108.00, 286.00]	167.00[78.00, 280.50]	0.146
PCT (μg/L)		2.49[0.67, 14.84]	1.06[0.39, 4.02]	4.06[0.74, 25.55]	<0.001
PT (S)		15.05[13.90, 17.90]	14.40[13.30, 15.95]	15.50[13.90, 18.70]	0.004
APTT (S)		36.35[32.15, 42.20]	32.70[29.15, 37.20]	37.00[33.95, 43.60]	<0.001
INR		1.22[1.12, 1.44]	1.16[1.08, 1.27]	1.26[1.14, 1.50]	0.002
ALT (U/L)		30.37[13.92, 72.42]	36.80[17.95, 58.15]	28.30[13.30, 74.70]	0.374
AST (U/L)		50.40[28.00, 106.39]	51.00[24.95, 90.35]	50.00[28.65, 132.00]	0.533
TBIL (μmol/L)		13.95[8.30, 26.10]	13.90[8.30, 22.10]	14.00[8.30, 26.90]	0.628
UN (mmol/L)		11.71[7.23, 18.94]	8.84[5.40, 15.42]	12.76[7.80, 20.30]	0.004
CR (μmol/L)		103.00[67.00, 157.65]	97.00[67.00, 136.71]	108.30[62.59, 170.00]	0.370
eGFR(ml/min/1.73m^2^)		60.37[36.23, 97.43]	68.15[43.86, 97.84]	58.68[32.08, 96.83]	0.228
OI		219.11[163.12, 294.50]	262.50[172.67, 322.16]	204.00[154.00, 283.33]	0.003
MAP		78.00[67.00, 85.00]	81.00[74.50, 85.00]	78.00[67.00, 84.00]	0.063
**Non-infectious treatment during hospitalization**					
Invasive respiratory mechanical ventilation		162.00(83.50%)	47.00(82.45%)	115.00(83.94%)	0.800
Vasoactive agents, n (%)		116.00(59.79%)	28.00(49.12%)	88.00(64.23%)	0.051
ECMO		4.00(2.03%)	0.00(0%)	4.00(2.92%)	0.454
CRRT		57.00(29.38%)	11.00(19.30%)	46.00(33.58%)	0.047
**Anti-infection program during hospitalization**					
Combined antifungal drugs		35.00(18.04%)	7.00(12.28%)	28.00(20.43%)	0.178
Antibiotic monotherapy		8.00(4.12%)	2.00(3.51%)	6.00(4.38%)	1.000
Types of combined antibiotics	MEM	52.00(26.80%)	17.00(29.82%)	35.00(25.55%)	
	IPM	79.00(40.72%)	17.00(29.82%)	62.00(45.25%)	
	TZP	25.00(12.88%)	12.00(21.05%)	13.00(9.49%)	
	POL	3.00(1.55%)	0.00(0%)	3.00(2.19%)	
	VOR	8.00(4.12%)	1.00(1.75%)	7.00(5.11%)	
	Others	27.00(13.92%)	10.00(17.54%)	17.00(12.41%)	
**Infection sites**					
Pulmonary infection		182.00(93.81%)	52.00(91.23%)	130.00(94.89%)	0.524
Septic shock		85.00(43.81%)	17.00(29.82%)	68.00(49.63%)	0.011
Bloodstream infection		34.00(17.52%)	8.00(14.03%)	26.00(18.98%)	0.409
Abdominal infection		37.00(19.07%)	11.00(19.29%)	26.00(18.98%)	0.959
Intestinal infection		8.00(4.12%)	0.00(0%)	8.00(5.84%)	0.142
Central nervous system infection		16.00(8.25%)	8.00(14.03%)	8.00(5.84%)	0.109
Skin and soft tissue infection		11.00(5.67%)	4.00(7.01%)	7.00(5.11%)	0.855
Urinary system infection		18.00(9.28%)	3.00(5.25%)	15.00(10.95%)	0.214
Myocarditis/endocarditis		8.00(4.12%)	1.00(1.75%)	7.00(5.11%)	0.500
Pancreatitis		3.00(1.55%)	2.00(3.51%)	1.00(0.73%)	0.429
**Previous history**					
Hospitalization history(<90d)		95.00(48.97%)	26.00(45.61%)	69.00(50.36%)	0.547
Surgery		60.00(30.93%)	16.00(28.07%)	44.00(32.12%)	0.579
History of antibiotics (<90d)		63.00(32.47%)	18.00(31.58%)	45.00(32.85%)	0.838
Immunosuppressive condition		4.00(2.06%)	0.00(0%)	4.00(2.92%)	0.450
**Combined underlying diseases**					
Hypertension		95.00(48.97%)	29.00(50.88%)	66.00(48.17%)	0.732
Hyperthyroidism		4.00(2.06%)	1.00(1.75%)	3.00(2.19%)	1.000
Hypothyroidism		1.00(0.52%)	1.00(1.75%)	0.00(0%)	0.650
Diabetes		38.00(19.59%)	9.00(15.79%)	29.00(21.17%)	0.114
Peptic ulcer		15.00(7.73%)	4.00(7.01%)	11.00(8.03%)	1.000
Chronic obstructive pulmonary disease/ asthma		18.00(9.28%)	4.00(7.01%)	14.00(10.22%)	0.484
Heart disease		44.00(22.68%)	7.00(12.28%)	37.00(27.01%)	0.026
Myocardial infarction		12.00(6.19%)	4.00(7.02%)	8.00(5.84%)	1.000
Congestive heart failure		21.00(10.82%)	2.00(3.51%)	19.00(13.87%)	0.034
Peripheral arterial disease		1.00(0.52%)	0.00(0%)	1.00(0.73%)	1.000
Cerebrovascular disease or TIA		40.00(20.62%)	10.00(17.54%)	30.00(21.89%)	0.495
Hemiplegia		3.00(1.55%)	1.00(1.75%)	2.00(1.46%)	1.000
Dementia/Alzheimer ‘s disease		2.00(1.03%)	1.00(1.75%)	1.00(0.73%)	1.000
Rheumatoid or connective tissue disease		3.00(1.55%)	0.00(0%)	3.00(2.19%)	0.626
Moderate/severe CKD		41.00(21.13%)	3.00(5.26%)	38.00(27.73%)	<0.001
Leukemia		3.00(1.55%)	1.00(1.75%)	2.00(1.46%)	1.000
Lymphoma		3.00(1.55%)	0.00(0%)	3.00(2.19%)	0.626
**Prognosis and outcome**					
ICU		176.00(90.72%)	54.00(94.73%)	122.00(89.05%)	0.214
Duration of ICU		17.37 ± 10.57	19.61 ± 9.66	16.44 ± 10.82	0.212
Outcome (0, 1 and 2)	0	87.00(44.84%)	31.00(54.38%)	56.00(40.87%)	<0.001
	1	30.00(15.46%)	3.00(5.26%)	27.00(29.71%)	
	2	76.00(39.17%)	23.00(40.35%)	53.00(38.68%)	
Hospital days		20.35 ± 9.77	21.17 ± 9.21	20.01 ± 10.02	0.365

**Note:** WBC: White blood cell count; PLT: Platelet count; OI: Oxygenation index; MAP: Mean arterial pressure; CR: Creatinine; TBIL: Total bilirubin; ALT: Alanine aminotransferase; AST: Aspartate aminotransferase; MEM: Meropenem; IPM: Imipenem; TZP: Piperacillin/Tazobactam; VOR: Voriconazole; POL: Polymyxin; PCT: Procalcitonin; APTT: Activated partial thromboplastin time; INR: International normalized ratio; UN: Urea nitrogen; OI: Oxygenation index; CRRT: Continuous renal replacement therapy; CKD: chronic kidney disease; 0 = cured/improved discharge,1 = died, 2 = gave up treatment and discharged automatically.

### Univariate analysis

Univariate analysis revealed statistically significant differences (P < 0.05) in procalcitonin (PCT), prothrombin time (PT), APTT, international normalized ratio (INR), UN, OI, continuous renal replacement therapy (CRRT), septic shock, heart disease, CHF and moderate/severe CKD between the vancomycin overexposure and compliant groups.

**Fig 1:**
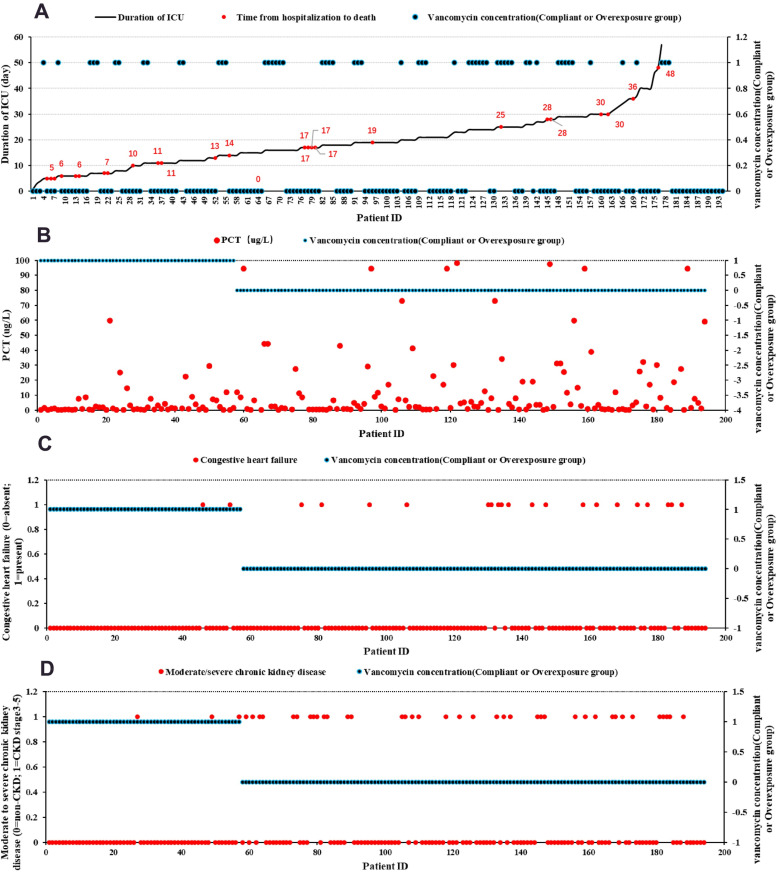
Visualization of critical clinical determinants. **(A)** Temporal dynamics of ICU duration (black solid line) versus time-to-mortality post-admission (red solid dots) and vancomycin compliant/overexposure curve (blue solid dots, 0 = Compliant group, 1 = Overexposure group). **(B)** The PCT trajectory (red solid dots) and vancomycin compliant/overexposure curve (blue solid dots, 0 = Compliant group, 1 = Overexposure group); **(C)** Congestive heart failure comorbidity status (red dots: 0 = absent, 1 = present) plotted against vancomycin exposure levels (blue solid dots, 0 = Compliant group, 1 = Overexposure group), highlighting cardiorenal toxicity associations. **(D)** Moderate/severe CKD progression (red dots: 0 = non-CKD, 1 = CKD stage 3-5) correlated with vancomycin exposure (blue solid dots, 0 = Compliant group, 1 = Overexposure group), quantifying nephrotoxicity risks across renal function strata.

To start with, as shown in Fig 1A, almost all patients who died during hospitalization occurred in the $C_{vg-min}$ overexposure group, while mortality in the $C_{vg-min}$ compliant group was not significant. Secondly, the patients of the $C_{vg-min}$ overexposure group generally had high PCT, which indicated more severe infection than the compliant group (4.06 vs 1.06, *P* < 0.001, Fig 1B). Thirdly, patients with CHF were more likely to occur in the $C_{vg-min}$ overexposure group compared with the compliant group (13.87% vs 3.51%, *P* = 0.034, Fig 1C). Fourthly, the patients with moderate/severe CKD were more likely to occur in the $C_{vg-min}$ overexposure group relative to the compliant group (27.73% vs 5.26%, *P* < 0.001, Fig 1D).

(**Note:** The data were analyzed by SPSS (IBM SPSS Statistics 21). Compliant group: 10μg/mL ≤ $C_{vg-min}$ ≤ 20μg/mL; Overexposure group: $C_{vg-min}$ > 20μg/mL).

### Multiple logistic regression

The factors satisfying *P* < 0.05 in univariate analysis were included in multivariate logistic regression analysis. After adjusting for confounding factors, the results showed that PCT, INR, UN, CRRT and septic shock had no significant effect on predicting the $C_{vg-min}$ overexposure (*P >* 0.05). There were significant differences in APTT, OI, CHF and moderate/severe CKD factors in predicting $C_{vg-min}$ was statistically different (*P* < 0.05) (Table 2), and they were more likely to induce $C_{vg-min}$ overexposure.

**Table 2:** Multivariate logistic regression analysis of factors affecting vancomycin serum trough concentration overexposure.

Variables	*OR*	95%*CI*	*P*-value
PCT	1.002	[0.996, 1.014]	0.637
APTT	1.052	[1.004, 1.110]	0.046
INR	1.496	[0.756, 4.626]	0.392
UN	1.027	[0.978, 1.085]	0.311
OI	0.995	[0.991, 0.999]	0.033
CRRT	0.964	[0.374, 2.486]	0.940
Congestive heart failure	5.023	[1.033, 24.430]	0.046
Moderate/severe CKD	5.187	[1.442, 18.663]	0.012
Septic shock	1.851	[0.845, 4.053]	0.124

**Note:** PCT: Procalcitonin; APTT: Activated partial thromboplastin time; INR: International normalized ratio; UN: Urea nitrogen; OI: Oxygenation index; CRRT: Continuous renal replacement therapy; CKD: chronic kidney disease.

### ROC curve analysis

**Fig 2:**
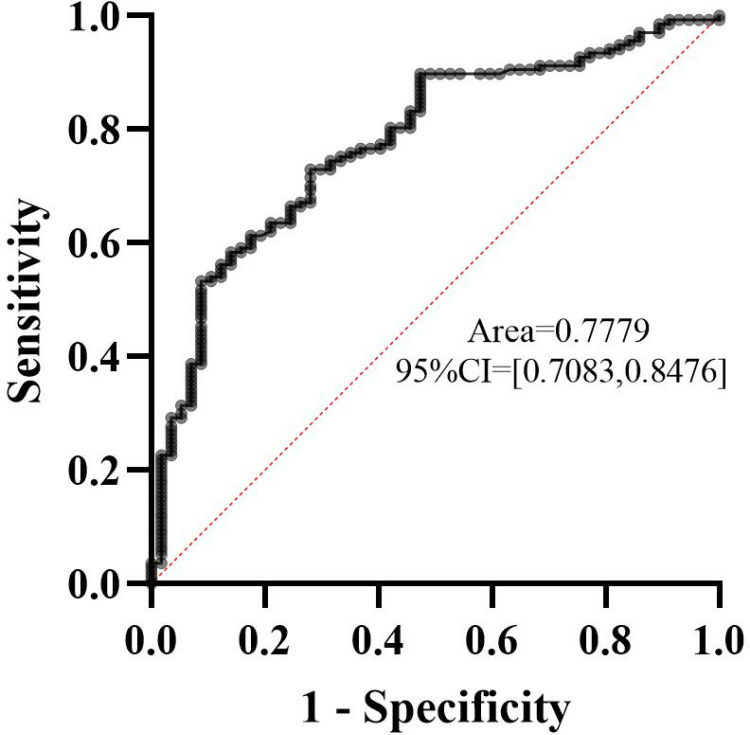
The ROC curve for APTT and OI to evaluate the accuracy of $C_{vg-min}$ **overexposure.**

The AUC corresponding to the ROC curve constructed for APTT and OI was 0.7779 (95% CI [0.708,0.848], *P* < 0.001). The sensitivity and specificity were 72.99% and 71.93%, respectively. The maximum Youden index of APTT was 0.370, and the optimal cutoff value of APTT was 33.65 s. When APTT > 33.65s, for every unit increase in APTT, the possibility of $C_{vg-min}$ overexposure increased by 1.052 times. The maximum value of the Youden index of OI was -0.2637, and the optimal cutoff value of OI was 245.5. When OI < 245.5, the risk of $C_{vg-min}$ exposure increased by 0.995 times. (Fig 2)

The overexposure rates of $C_{vg-min}$ in patients with moderate/severe CKD and CHF were 39/41(95.12%) and 20/21(95.23%), respectively. With regard to OI, when the cutoff value of OI was less than 245, the $C_{vg-min}$ overexposure rate was 93/112 (83%); otherwise, the overexposure rate was 50/82(60.97%). For APTT, the overexposure rate was 107/131(81.67%) when the cutoff value of APTT was more than 33.65 s; otherwise, the overexposure rate was 32/63(50.79%).

### Principle of AHP

Based on drug labeling and literature analyzing the adverse drug reactions induced by three types of anti-MRSA drugs[23]. According to the idea of AHP and Saatty criterion, the weight judgment matrix of these factors on $C_{vg-min}$ was obtained, and the judgment matrix A of the criterion layer is as follows.


$$\text{A= }[\begin{array}{cc} \;\begin{array}{cc} 1\; & \;1/3\\ 3\; & \;1 \end{array}\; & \;\begin{array}{cc} 1/5 & \;1/7\\ 2 & \;1/4 \end{array}\;\\ \begin{array}{cc} 5\; & \;1/2\\ 7\; & \;4 \end{array} & \;\begin{array}{cc} 1\; & 1/5\\ 5\; & 1 \end{array}\; \end{array}]$$

Based on https://clincalc.com/Vancomycin/ and JPKD software, and the weight analysis of three dosing regimens (increasing dose, delaying infusion time and increasing the dosing interval) on APTT, OI, CHF and moderate/severe CKD factors. The judgment matrix of the scheme layer is shown in Table 3.

**Table 3:** Scheme layer judgment matrix.

B1	C1	C2	C3	B2	C1	C2	C3	B3	C1	C2	C3	B4	C1	C2	C3
C1	1	7	7	C1	1	7	7	C1	1	7	7	C1	1	7	7
C2	1/7	1	1/2	C2	1/7	1	1/2	C2	1/7	1	1/2	C2	1/7	1	1/2
C3	1/7	2	1	C3	1/7	2	1	C3	1/7	2	1	C3	1/7	2	1

The weights of APTT, OI, CHF and moderate/severe CKD to the overexposure of $C_{vg-min}$ were 0.0584, 0.1899, 0.1614 and 0.5902, respectively. The *CI* and *CR* were 0.0796 and 0.0885, which meet the consistency test standard. It shows that the hierarchical total ranking results achieve satisfactory consistency and the analysis results were accepted. The ranking of the influencing factors was as follows: Moderate/severe CKD > OI > CHF > APTT. According to the definition of combination weight vector, the combination weight vector *w* = (0.7671 0.09 0.1429) was obtained by substituting the data, meaning that the contribution weights of increasing dose, increasing the dosing interval and delaying infusion time to the overexposure of $C_{vg-min}$ were 0.7671, 0.09 and 0.1429, respectively. The combination *CI* and *CR* were 0.0272 and 0.0469, separately, which meet the consistency test standard. Based on these results, the hierarchical total ranking results have satisfactory consistency and the analysis results are accepted. The effects of different dosing regimens on the vancomycin overexposure were ranked as follows: increasing dose > delaying infusion time > increasing the dosing interval.

## Discussion

The present study enrolled 194 critically ill patients with sepsis or septic shock according to the definition by the third international consensus [24], and international guidelines for management of sepsis and septic shock [25]. Tobias Zimmermann et al conducted a retrospective study of gender differences in sequential organ failure assessment (SOFA) scores in patients with sepsis or septic shock in the intensive care unit, which revealed the existence of sex-specific differences in the SOFA score of patients admitted due to sepsis or septic shock [26]. In addition, Emma Larsson mentioned that animal models showed that females were less susceptible to sepsis and tended to recover more effectively than males [27]. The different responses of female and male hosts to pathogens can be partially attributed to the sex-specific polarization of intracellular pathways that respond to pathogen-cell receptor interactions. Therefore, it may be used to explain the baseline phenomenon of gender ratio in our study.

OI is also mentioned as PaO2/FiO2, which is commonly used to measure the severity of hypoxemia in patients with respiratory failure. The importance of oxygen shows that the cells need oxygen to maintain survival and function, and hypoxia leads to irreversible damage to important organs. Whether it is ventilation dysfunction or ventilation dysfunction, oxygen can’t be transported normally in the body, resulting in hypoxia. Through the oxygen delivery (DO2) formula [DO2 = arterial oxygen content(CaO2)×cardiac output(CO)×10, CaO2 = hemoglobin (Hb)×1.34 × arterial oxygen saturation (SaO2)+partial pressure of arterial oxygen (PaO2)×0.0076}, it can be seen that the ventilation and ventilation function of the lung and the cardiac output of the heart are involved in the occurrence of hypoxemia during oxygen delivery [28]. Therefore, one possible explanation for $C_{vg-min}$ overexposure is the hypoxia-induced impairment of drug metabolism in patients with low OI, causing damage to the corresponding muscle cells. For another, H Wang et al showed that OI was an independent risk factor for intra-abdominal hypertension [29]. Additionally, it has been mentioned that intra-abdominal hypertension is not uncommon in critically ill patients, and its incidence can reach 30% ~ 40% [30]. The increased intra-abdominal pressure can affect systemic hemodynamics[10], which may affect renal blood flow and result in the increased vancomycin concentration. In this study, OI as one of the risk factors for the excessive exposure of $C_{vg-min}$ may be due to the increase in intra-abdominal pressure caused by hypoxia index, which affects the changes of renal blood flow.

CKD is defined as renal structural or functional abnormalities for more than 3 months [31]. For CKD patients, not only the renal clearance rate decreases, but also the liver drug enzyme activity decreases from 5% to 50% [32]. In our study, there was statistically significant difference in the compliant group (5.26%) compared with the overexposure group (27.73%) in patients with moderate/severe CKD (*P* < 0.001). Through the multivariate logistic regression and AHP analyses, moderate/severe CKD was one of the risk factors leading to $C_{vg-min}$ overexposure. In addition, it is reported that the risk of infection in CKD patients is 3 ~ 4 times higher than that in normal people [33]. Further, $C_{vg-min}$ values over 21.5 mg/L and 16.5 mg/L are associated with an increased risk of vancomycin-induced nephrotoxicity in CKD Stage 3a and 3b-5 [34], respectively. Similar to these studies, our results suggested that kidney disease might affect $C_{vg-min}$ overexposure.

CHF is generally defined as the inability of the heart to supply sufficient blood flow to meet the needs of the body [35]. Due to the decreased cardiac output and the decreased renal blood flow, CHF can alter the pharmacokinetics of various drugs. In our study, through multivariate logistic regression analysis of factors affecting $C_{vg-min}$ overexposure, the results showed that the OR and 95%CI for CHF factor were 5.023 and 1.033 ~ 24.430 (*P* = 0.046). This indicates that CHF may be another risk factor leading to $C_{vg-min}$ overexposure, which can be explained by the relevant literature. Shammas et al showed the major influences of CHF on drug pharmacokinetics, which were duction in the volume of distribution and an impairment of elimination clearance, and consequently a prolonged elimination half-life [35]. Yuko Shimamoto et al summarized that vancomycin clearance, which was affected by cardiac function, decreased with the decreasing cardiac function and the decreasing creatinine clearance [36].

APTT is a screening test for endogenous coagulation factors. The prolongation of APTT is common in coagulation factor deficiency, and its shortening indicates that the blood is in a hypercoagulable state. The laboratory examination of DIC includes PT, APTT, fibrinogen concentration and platelet count that reflect the consumption of coagulation factors [37]. Clinically, the incidence of DIC is relatively high in critically ill patients. DIC symptoms occur in 10% ~ 30% of critically ill patients, which complicates the clinical course and increases the mortality of patients [38]. Besides, infection is the most important cause of DIC, and 30% ~ 51% of infected patients may develop the DIC symptoms [39]. Our study included 194 critically ill patients. There were statistical differences in PCT, PT, APTT and INR between the compliant and overexposure groups (*P* < 0.05). DIC can cause the body coagulation-anticoagulation-fibrinolysis system disorders, massive microvascular thrombosis, and ultimately induce bleeding, multiple organ failure and other symptoms, which may alter the vancomycin pharmacokinetics. Therefore, the reason for the increase of $C_{vg-min}$ in critically ill patients with DIC may be related to the multi-organ failure caused by DIC. Through the multiple logistic regression and AHP, APTT may be another risk factor leading to $C_{vg-min}$ overexposure.

**Fig 3:**
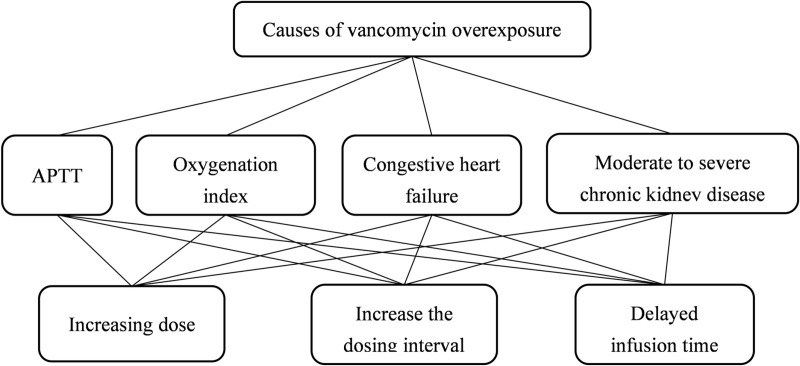
Flow chart of the AHP.

In the present study, with the reason of $C_{vg-min}$ overexposure being the target layer, and the factors after multiple logistic regression being the criterion layer, the AHP model was constructed (Fig 3). The hierarchical total ranking results had satisfactory consistency and thus were accepted (*CR *= 0.0469 < 0.1). The ranking of the influencing factors was as follows: Moderate/severe CKD > OI > CHF > APTT. And the scheme layer sorting was ranked as follows: increasing dose > delaying infusion time > increasing the dosing interval. Therefore, if excessive $C_{vg-min}$ was exposed, the vancomycin dosing regimen could be adopted as follows: reducing the dosage > shortening the infusion time (more than 60 min of infusion according to the drug instructions)> reducing the dosing interval.

## Conclusions

Our investigation systematically reveals the clinical prioritization of factors influencing $C_{vg-min}$ overexposure in mechanically ventilated critically ill patients. The derived clinically significant ranking demonstrates that moderate/severe CKD is the predominant determinant, followed by OI impairment, CHF and APTT alterations. This evidence-based hierarchy provides crucial insights for clinical decision-making regarding the risk stratification and intervention prioritization in the critical care settings. However, these findings should be interpreted with caution due to the limitations of this study, including its single-center design and relatively small sample size. Future multi-center studies with larger cohorts are warranted.
